# Ricinine toxicity in susceptible lepidoptera involves perturbations in *β*-nicotinamide adenine dinucleotide metabolism

**DOI:** 10.3389/fphys.2026.1947596

**Published:** 2026-09-11

**Authors:** Qin Li, Ru-yi Jin, Chao-wei Wen, Hao-yang Ma, Zuo-min Shao, Xue-yang Wang, Li-shang Dai, Lu-feng Hu, Ming-jie Deng

**Affiliations:** 1 Jiangsu Key Laboratory of Sericultural Biology and Biotechnology, School of Biotechnology, Jiangsu University of Science and Technology, Zhenjiang, China; 2 Department of Pharmacy, The First Affiliated Hospital of Wenzhou Medical University, Wenzhou, China; 3 School of Laboratory Medicine and Life Sciences, Wenzhou Medical University, Wenzhou, China; 4 School of Pharmaceutical Sciences, Wenzhou Medical University, Wenzhou, China

**Keywords:** *Bombyx mori*, interaction mechanism, ricinine, *Samia cynthia ricini*, *Spodoptera litura*, β-NAD

## Abstract

Ricinine, a plant-derived alkaloid, exhibits potent insecticidal activity; however, the molecular mechanisms underlying its differential toxicity among insect species remain largely elusive. This study employed integrated metabolomics and transcriptomics to investigate the adaptation strategies of three lepidopteran insects with distinct host plant ranges—*Bombyx mori* (*B. mori*), *Spodoptera litura* (*S. litura*), and *Samia cynthia ricini* (*S. cynthia ricini*) to ricinine. The three species exhibited sharply divergent toxicological responses: ricinine caused acute lethality in *B. mori*, severely inhibited the growth of *S. litura*, but induced no significant adverse effects in *S. cynthia ricini*. Untargeted metabolomics and transcriptomics revealed that ricinine treatment provoked the most severe metabolic disruption in *B. mori*, followed by *S. litura*, while the impact on *S. cynthia ricini* was minimal. Moreover, the species showed largely species-specific pathway-enrichment patterns, indicating fundamentally different adaptive mechanisms. Integrated analysis identified decreased normalized relative abundances of key metabolites (e.g., adenine, flavin mononucleotide) and significant downregulation of genes associated with NAD/NADH-linked energy metabolism (e.g., *nicotinate phosphoribosyltransferase* (*NPH*), *malate dehydrogenase* (*MDH*), *acetyl-CoA synthetase* (*ACS*)) in susceptible species. These molecular changes were associated with *β*-nicotinamide adenine dinucleotide (*β*-NAD) metabolism and oxidative phosphorylation-related pathways. Exogenous *β*-NAD supplementation alleviated ricinine toxicity in *S. litura*, supporting the involvement of *β*-NAD-related metabolism. In contrast, *S. cynthia ricini*, despite accumulating the highest levels of ricinine, displayed only marginal metabolic and transcriptional perturbations, signifying the evolution of robust innate tolerance mechanisms. Finally, the combined treatment with ricinine and nucleopolyhedrovirus (NPV) produced greater growth inhibition and mortality in *S. litura* than either treatment alone, which was associated with the marked inhibition of cytochrome P450 detoxification enzyme activity. These findings elucidate evolutionary strategies insects employ to cope with plant alkaloids and provide a molecular basis for developing novel, selective pest control agents.

## 1 Introduction

Through long-term co-evolution with insects, plants have developed a sophisticated suite of chemical defenses, which include defensive proteins and a diverse range of secondary metabolites (Konno, 2011). Alkaloids, in particular, are recognized for their significant role in plant defense against insects. They function not only as defensive compounds that deter herbivory but also as mediators of chemical communication between plants and insects (Rebholz et al., 2023). In response, insects have evolved counter-adaptations, among which the sequestration of plant defensive compounds is a key strategy that provides a selective advantage (Beran and Petschenka, 2022), while species-specific metabolic responses to ricinine remain understudied. This phenomenon of sequestration is a central component of the ongoing arms race between plants and insects.

Among these plant defenses, ricinine, a pyridine alkaloid from the castor bean plant (*Ricinus communis*), is well-known for its potent insecticidal properties. However, the molecular mechanisms underlying its toxicity and the reasons for the vast differences in susceptibility among insect species remain poorly understood. A fascinating natural experiment exists in *S. cynthia ricini*, a silkworm that has specialized on feeding on castor bean leaves, implying the evolution of a powerful resistance or tolerance mechanism to ricinine. Comparing the response of this specialist with susceptible species offers a unique opportunity to dissect the physiological and genetic basis of adaptation to plant toxins.

Ricinine has garnered significant attention due to its diverse biological activities and potential applications. This compound has been found to possess a wide range of pharmacological properties, including anti-inflammatory, antioxidant, cytotoxic and pesticidal effects (Khan et al., 2016; Towle et al., 2026). The toxicity of ricinine extends beyond its inhibition of protein synthesis to encompass multifaceted effects across diverse organisms. In animal models, ricinine inhalation induces localized inflammatory responses, accompanied by pulmonary congestion, edema, neutrophil infiltration, and severe acute respiratory distress syndrome (Roy and Ehrbar, 2022). Notably, ricinine toxicity exhibits variability across animal models, which may correlate with factors such as exposure methods, aerosol characteristics, toxin purity, and challenge doses (Stoll et al., 2023). These findings demonstrate that ricinine toxicity depends not only on its chemical properties but also on the physiological and immune responses of the host organism. In humans, ingestion of ricin-containing castor beans may cause severe gastrointestinal symptoms, dehydration, gastrointestinal bleeding, hypotension, and, in severe cases, progressive multiorgan failure (Rauniyar et al., 2025). This mechanism has made ricinine a potential bioterrorism weapon, as its high toxicity and easy availability have led to its repeated use for malicious purposes.

However, studies on the natural resistance of organisms to ricinine remains limited, and the available evidence is fragmented. Although significant progress has been made in ricinine–vaccine development, several challenges remain. For instance, the correlation between serum antibody titers and actual protective efficacy remains unclear, complicating the evaluation of vaccine efficacy. Elucidating the molecular basis of interspecific sensitivity differences to ricinine may facilitate the development of novel detoxification strategies. Through long-term evolution, the larvae of *S. cynthia ricini* have evolved adaptive or avoidance mechanisms against ricinine, enabling them to feed on castor leaves. This makes *S. cynthia ricini* an excellent model for studying ricinine-host interactions, thereby facilitating study on the mechanism of action of ricinine in *S. cynthia ricini* or other lepidopteran insects.

To bridge this knowledge gap, this study employed an integrated untargeted metabolomics and transcriptomics approach to comparatively investigate the responses of three lepidopteran insects: the highly susceptible *B. mori*, the moderately susceptible pest *S. litura*, and the highly tolerant specialist *S. cynthia ricini*. We aimed to characterize the species-specific phenotypic and physiological effects of ricinine, identify the key metabolic pathways and gene networks perturbed by ricinine, and elucidate the distinct adaptive strategies, from metabolic vulnerability to evolved tolerance, that underlie their divergent responses. This comparative framework is designed to provide fundamental insights into the evolutionary biology of insect adaptation to plant chemical defenses.

## 2 Materials and methods

### 2.1 Insect breeding

Larvae of *S. litura* were obtained from Keyun Biological Co., Ltd. (Jiyuan, China) and reared on a standard artificial diet following (Dai et al., 2021). Larvae of *S. cynthia ricini* and *B. mori* were provided by the Sericultural Research Institute, Chinese Academy of Agricultural Sciences at Jiangsu University of Science and Technology (Zhenjiang, China). *S. cynthia ricini* and *B. mori* larvae were fed artificial diets containing cassava leaf powder and mulberry leaf powder, respectively. Importantly, none of the diets contained ricinine to preclude any unintended physiological effects from this potent toxin. All insects were maintained under controlled conditions: 26 °C, 60–80% relative humidity, and a 12-h light/12-h dark photoperiod.

### 2.2 Toxicology assay

To investigate the toxic effects of ricinine (supplied by Shanghai Yuanye Bio-Technology Co., Ltd., Shanghai, China) toxin on lepidopteran insects, we employed 2nd to 3rd instar larvae. Within each species, larvae were synchronized by developmental stage and individuals at the same instar were randomly assigned to the experimental groups. The larvae were randomly allocated into three treatment groups with 10 individuals per group: each replicate consisted of a separate cohort of 10 larvae and was considered an independent biological replicate (30 larvae per treatment in total). For each experimental group, a separate biological replicate group with the same number of larvae was set up. Biological replicate refers to independently treated individuals or groups, and technical replicate refers to multiple measurements of the same sample. The treatment groups were: (1) a control group fed a standard diet; (2) a low-dose group fed a diet supplemented with 0.2% (w/w) ricinine toxin; and (3) a high-dose group fed a diet supplemented with 0.5% (w/w) ricinine toxin. Ricinine was incorporated directly into the artificial diet without an organic solvent or other delivery vehicle. The control diet was prepared following the same protocol as the treatment diet, excluding ricinine. Therefore, a separate solvent-control group was not required. The control diet was prepared and handled identically to the treatment diets but contained no ricinine. All groups were maintained under identical environmental and feeding conditions. The body weight of each larva was recorded daily to assess growth and toxic responses. To account for interspecific differences in initial body size and growth rate, growth responses were expressed relative to the initial body weight and the corresponding time-matched control of the same species. Raw body weights were not directly compared among species. The inhibiting rates were calculated by formula sa below.


$$\text{Inhibiting rate}=\left(1-\frac{\text{average weight of experimental group}}{\text{average weight of Control}}\right)\times 100\%$$

### 2.3 Sample collection for metabonomics and transcriptomics

The hemolymph from developmentally matched 5th-instar larvae of *S. litura*, *S. cynthia ricini*, and *B. mori* was employed to investigate metabolic alterations induced by ricinine ingestion, with sampling based on instar stage rather than chronological age to ensure physiological comparability. Sixteen 5th-instar larvae of each species were randomly allocated into two groups (n = 8 biologically independent larvae per treatment per species): one received an artificial diet supplemented with 0.5% ricinine, while the control group was maintained on a ricinine-free diet. All samples were collected 48 h after treatment at the same instar within each species. Because absolute developmental rates and physiological baselines differ among species, treatment effects were evaluated relative to the matched control of the same species rather than by directly comparing raw measurements among species. Hemolymph from each larva was collected, processed, and analyzed as an individual biological sample without pooling; LC-MS and RNA sequencing (RNA-Seq) measurements were performed once per biological sample, and no technical measurement was treated as an independent replicate. After 48 h of feeding, hemolymph was collected from all individuals and immediately frozen at -80 °C for subsequent high-performance liquid chromatography-mass spectrometry (HPLC-MS). For transcriptome analysis, each hemolymph sample (50 μL) was homogenized with 150 μL of TRIzol reagent (Invitrogen, Grand Island, NY, USA).

### 2.4 HPLC-MS analysis of three insect hemolymph samples

Non-targeted metabolomic analysis was conducted using a liquid chromatography-tandem mass spectrometry (LC-MS/MS) system (Thermo Fisher Scientific). Metabolite identification and quantification were performed with MS-DIAL software against multiple MS/MS spectral libraries. Specifically, hemolymph (50 μL) was transferred to a 2 mL centrifuge tube. Extraction solvent (1 mL of methanol:acetonitrile:water, 2:2:1, *v*/*v*/*v*) and two glass beads were added, followed by vortexing for 30 s. The mixture was flash-frozen in liquid nitrogen for 5 min, thawed at room temperature, and homogenized at 55 Hz for 60 s using a high-throughput tissue homogenizer. This freeze-thaw-homogenization cycle was repeated twice. After centrifugation (12,000 × g, 15 min, 4 °C), the supernatant (850 μL) was collected and dried under vacuum. The residue was reconstituted in 150 μL of 50% methanol containing 5 ppm 2-chloro-L-phenylalanine as an internal standard, followed by vortexing for 30 s. After a second centrifugation (12,000 × g, 10 min, 4 °C), the supernatant was filtered through a 0.22 μm membrane into an autosampler vial for LC-MS/MS analysis.

Chromatographic separation was performed on an ACQUITY UPLC HSS T3 column (100 Å, 1.8 μm, 2.1 mm × 100 mm) maintained at 40 °C. The mobile phase consisted of (A) 0.1% ammonium hydroxide in water and (B) acetonitrile with 0.1% formic acid. The gradient elution program was: 0.0–1.0 min, 5% B; 1.0–4.7 min, 5%–95% B; 4.7–6.0 min, 95% B; 6.0–6.1 min, 95%–5% B; 6.1–8.5 min, 5% B. The flow rate was 0.4 mL/min, the injection volume was 2 μL, and the autosampler temperature was maintained at 8 °C.

Mass spectrometry was performed on a Thermo Orbitrap Exploris 120 instrument controlled by Xcalibur software (v4.7). A heated electrospray ionization (HESI) source was operated with spray voltages of 3.5 kV (positive) and −3.0 kV (negative). Sheath and auxiliary gas flow rates were set to 40 and 10 arbitrary units, respectively. The capillary and auxiliary gas heater temperatures were maintained at 320 °C and 300 °C, respectively. Full-scan MS data were acquired at a resolution of 60,000 over m/z 70–1000, with an automatic gain control (AGC) target of ‘Standard’ and a maximum injection time of 100 ms. Data-dependent MS/MS acquisition was performed on the top four most intense ions, using higher-energy collisional dissociation (HCD) with 30% normalized collision energy. MS/MS spectra were collected at a resolution of 15,000, with a dynamic exclusion of 4 s.

Raw data files (.raw) were processed using MS-DIAL (version 4.9.221218) for peak detection, alignment, and filtering, missing-value imputation, normalization, and metabolite annotation. Features with more than 50% missing values in every experimental group were excluded. The principal MS-DIAL parameters were as follows: MS1 tolerance for identification, 0.01 Da; MS2 tolerance for identification, 0.05 Da; smoothing level, 3; minimum peak height, 10,000; minimum peak width, 5; mass slice width, 0.05; and identification score cut-off, 70. Metabolites were identified by detected accurate-mass and MS/MS spectra against the PerSonalbio next-generation metabolomics database (PSNGM Database), which integrates an in-house authentic-standard library, mzCloud, LIPID MAPS, HMDB, MoNA, NIST_2020_MSMS, and an AI-predicted MS/MS spectral library. MS-DIAL integrates the principal factors affecting annotation reliability into a Total Score, which is reported in the metabolite-identification table. Only compounds with a Total Score ≥70 and an identification confidence of Level 2 or higher were retained. A higher Total Score indicates greater confidence in the metabolite annotation. The detection limit (LOD) of this non-targeted metabolomics method is approximately 0.01 μM, and the quantification limit (LOQ) is approximately 0.05 μM (estimated based on the internal standard response).

### 2.5 RNA extraction, transcriptome sequencing, and functional analysis

Total RNA was extracted using Trizol reagent (Invitrogen Life Technologies, China), and its quality was assessed using a NanoDrop microspectrophotometer (Thermo Scientific). Three micrograms of qualified RNA were used for subsequent library preparation.

The sequencing library was constructed following these steps: messenger RNA (mRNA) was first isolated from total RNA using oligo(dT)-coupled magnetic beads. Fragmentation was then performed under high-temperature conditions in the presence of divalent cations using Illumina’s proprietary fragmentation buffer. First-strand complementary DNA (cDNA) synthesis was carried out with random hexamer primers and SuperScript II reverse transcriptase, followed by second-strand synthesis using DNA Polymerase I and RNase H. The resulting DNA fragments were blunt-ended through exonuclease/polymerase treatment, and residual enzymes were removed. After adenylation of the 3’ ends, Illumina PE adapter oligonucleotides were ligated. Library fragments of 400–500 bp were size-selected using the AMPure XP system (Beckman Coulter, Beverly, CA, USA). Adapter-ligated fragments were enriched by 15 cycles of polymerase chain reaction (PCR) with Illumina PCR primers, and the final library was purified and quantified using the Agilent Bioanalyzer 2100 system with the High Sensitivity DNA assay. The completed library was sequenced on an Illumina NovaSeq 6000 platform.

Sequencing generated raw image files that were converted to FASTQ format using the Illumina basecalling pipeline. Differential gene expression analysis was performed with DESeq2 (v1.38.3). Raw read counts were modeled using a negative-binomial generalized linear model, with library-size differences normalized using the DESeq2 median-of-ratios method. For each species, ricinine-treated and control samples were compared using the Wald test. The resulting *p*-values were corrected for multiple testing using the Benjamini–Hochberg procedure, and genes with |log_2_FoldChange| >1 and an adjusted *p*-value (false-discovery rate, FDR)<0.05 were considered differentially expressed. Additional analyses including expression profiling, exon usage, and functional enrichment were conducted using the Personalbio GenesCloud platform (www.genescloud.cn).

Because a reference genome is unavailable for *S. ricini*, clean reads were assembled *de novo* using Trinity v2.15.1, which reconstructs transcripts based on a de Bruijn graph algorithm. The resulting unigenes were subjected to functional annotation. Sequence similarity searches against the NCBI non-redundant protein (NR) database were performed using the BLASTX mode of DIAMOND v0.9.25.126. Gene Ontology (GO) terms and eggNOG orthology annotations were assigned using eggNOG-mapper v2.1.6, whereas Kyoto Encyclopedia of Genes and Genomes (KEGG) annotations were obtained using KOBAS v3.0.

### 2.6 Reverse-transcription PCR (RT-qPCR)

To determine the relative expression levels of target genes, RT-qPCR was performed. Gene-specific primers for *S. litura* and *B. mori* were designed using the PrimerQuest™ Tool (Integrated DNA Technologies, https://sg.idtdna.com). All primer sequences are listed in **Supplementary Table 1**. All oligonucleotide primers were synthesized by Sangon Biotech (Shanghai, China). Total RNA was isolated from hemolymph samples using TRIzol reagent (Invitrogen, China). Subsequently, 2 μg of total RNA were reverse-transcribed into cDNA using the HiScript III RT SuperMix for qPCR (Vazyme, China). RT-qPCR reactions were carried out using ChamQ Universal SYBR qPCR Master Mix (Vazyme) on a CFX96 Touch Real-Time PCR Detection System (Bio-Rad, USA). The cycle threshold (Ct) values were determined, and relative gene expression levels were calculated using the comparative 2^−ΔΔCT^ method. All experiments included three independent biological replicates, each derived from an independently collected larval hemolymph sample. Each biological replicate was analyzed in three technical wells, and the mean Ct value of the technical triplicates was used as one biological observation. Technical replicates were not treated as independent samples in the statistical analysis. Data are expressed as the mean ± standard error of the mean (SEM).

### 2.7 Data refinement and diversified pattern identification analysis

The identified metabolites (from both negative and positive ion modes) and their corresponding peak area data were imported into Excel. For each sample, the detected peak areas were normalized to a total signal of 10,000, and the resulting values were used as the normalized relative abundances of the corresponding metabolites. Accordingly, the LC-MS untargeted metabolomics analysis provided relative rather than absolute quantification. The normalized values were imported into SIMCA-P 13.0 software (Umetrics, Umeå, Sweden) for pattern recognition. Principal component analysis (PCA) was performed separately for each species to compare ricinine-treated larvae with their species-matched controls. For differential-metabolite analysis, treatment effects were expressed as within-species fold changes relative to the corresponding control. Raw metabolite intensities were not used for direct cross-species comparisons because baseline metabolite abundance and matrix effects may differ among species. PCA was used as an unsupervised exploratory method and was not used to determine statistical significance. For each insect species, normalized metabolite abundances were compared between the ricinine-treated and control groups using Welch’s two-sided t-test. To account for simultaneous testing of multiple metabolites, the resulting *p*-values were adjusted using the Benjamini–Hochberg procedure. Metabolites with an FDR-adjusted *p*-value (q-value)<0.05 were considered statistically significant. Based on the PCA results, group separation was visualized, whereas statistical inference was based on the FDR-adjusted univariate comparisons described above.

### 2.8 Analysis of NAD on the toxicity of ricinine to *B. mori* and *S. litura* larvae

To confirm the role of *β*-NAD (Shanghai Yuanye Bio-Technology Co., Ltd. China) in insects following ricinine expose, an exogenous *β*-NAD supplementation assay was conducted using thirty larvae each of *B. mori* and *S. litura*. For each species, the experiment comprised three independent biological replicates, with ten larvae per treatment in each replicate. A separate cohort of larvae was used for each biological replicate; individual larvae were the experimental units, and body weight and mortality were measured once at each time point without technical replication. Initially, all larvae were weighed. Each species was then maintained on an artificial diet and assigned to one of three treatment groups: (1) Control: standard diet; (2) Ricinine: diet containing 0.5% ricinine; (3) Ricinine + *β*-NAD: diet containing 0.5% ricinine supplemented with 0.5% *β*-NAD (Yuanye Bio-Technology Co., Ltd., Shanghai, China).

### 2.9 Bioassay of ricinine in combination with autographa californica multiple nucleopolyhedrovirus

To explore the effect of combining ricinine with AcMNPV, the combined treatment effect of ricinine and AcMNPV against the tobacco cutworm *S. litura* was analyzed. Wild-type AcMNPV budded virus (BV) was maintained by the Key Laboratory of Silkworm and Mulberry Genetic Improvement, School of Biotechnology, Jiangsu University of Science and Technology. The virus titer was propagated in Sf9 cells, and its titer was determined using a standard curve method. A total of 180 *S. litura* larvae were divided into four groups: control, ricinine treatment alone, AcMNPV infection alone, and combined ricinine + AcMNPV treatment, with three biological replicates per group. Each biological replicate consisted of an independently treated cohort of 15 larvae. Individual larvae were the experimental units for body-weight and survival analyses, whereas cytochrome P450 (CYP450) activity was measured from independently collected biological samples; assay readings from the same sample were treated as technical replicates and were averaged before statistical analysis. Larvae were treated with 0.2% ricinine starting from the early third instar. After 48 h, each larva was inoculated with 2 μL of 1×10^5^ plaque-forming units per milliliter (PFU/mL) AcMNPV. Subsequently, larval body weight and mortality were recorded daily, and cytochrome P450 enzyme activity was measured. Enzyme activity was determined using a CYP450 activity assay kit (Yuanye Bio-Technology Co., China), following the manufacturer’s instructions. Equal volumes of hemolymph were assayed for all samples, and CYP450 activity was calculated from the kit standard curve and expressed as ng/mL of hemolymph.

### 2.10 Statistical analysis

Statistical analyses were performed using SPSS 17.0 (SPSS Inc., Chicago, IL, USA). The statistical model was selected according to the experimental design, distribution of the response variable, and homogeneity of variance. All the data are first subjected to the Shapiro-Wilk normality test; if the data conform to a normal distribution and the variances are homogeneous (as tested by the Levene test), then a one-way ANOVA is used; otherwise, the Kruskal-Wallis test is employed. Upon obtaining a significant ANOVA result, Tukey’s *post hoc* test was applied for multiple comparisons. Biological replicates, rather than technical measurements, were used as the units of statistical inference. All data are presented as the mean ± standard deviation. A *p*-value of less than 0.05 was considered statistically significant.

## 3 Results

### 3.1 Toxicity tests of ricinine on *B. mori*, *S. cynthia ricini*, and *S. litura* larvae

**Figure 1:**
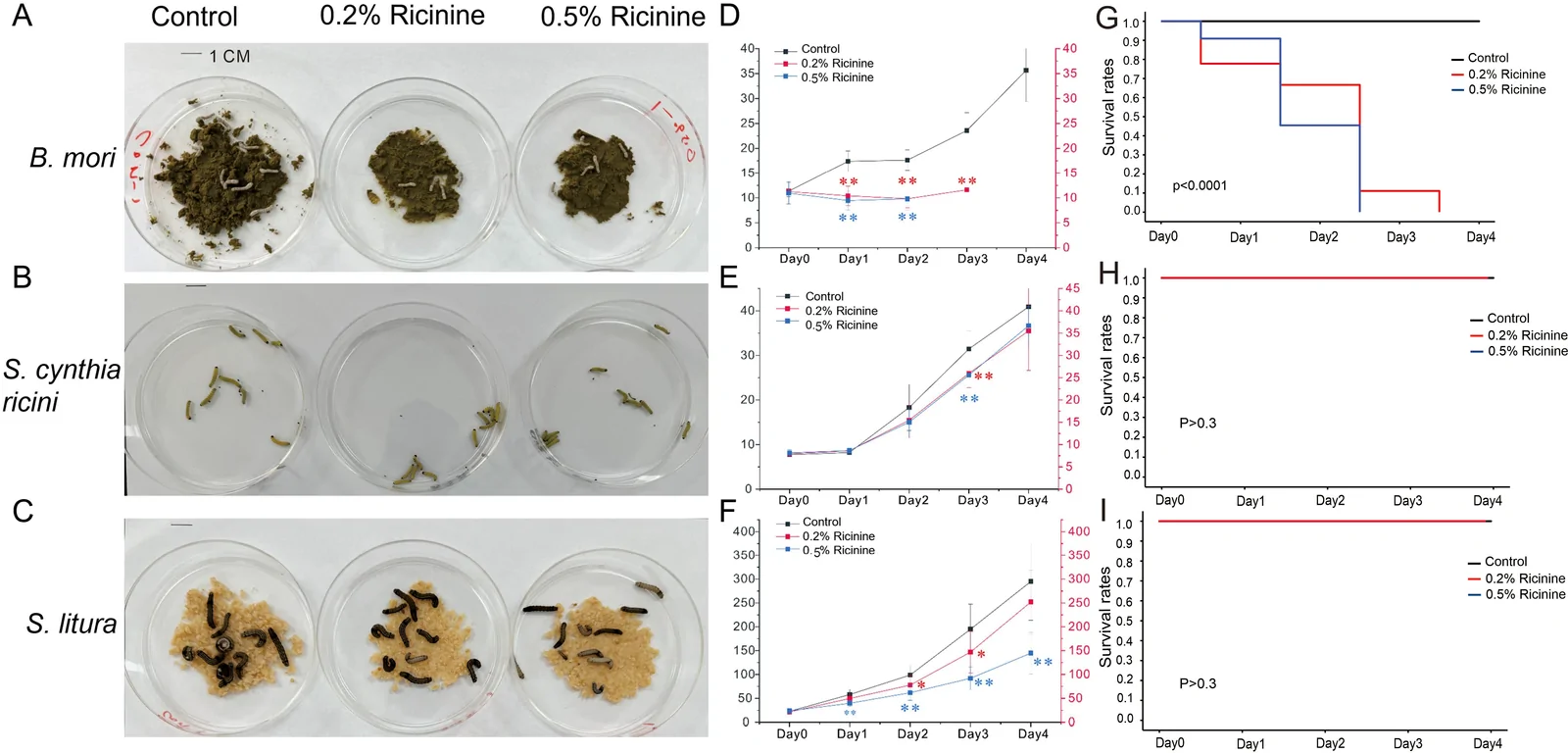
Larval growth dynamics of *B. mori*, *S. cynthia ricini*, and *S. litura* larvae following exposure to different doses of ricinine over time. **(A–C)** Morphological phenotypes of *B. mori*, *S. cynthia ricini*, and *S. litura* larvae treated with 0.2% and 0.5% ricinine on day 2. **(D–F)** Body weight measurements of *B. mori*, *S. cynthia ricini*, and *S. litura* larvae after exposure to 0.2% and 0.5% ricinine for different durations. For **(D–F)**, the x-axis indicates time after treatment, and the y-axis indicates larval body weight (mg). Black, red, and blue symbols represent the control, 0.2% ricinine, and 0.5% ricinine groups, respectively. **(G–I)** The Log-rank survival curves of *B. mori, S. cynthia ricini*, and *S. litura* larvae after exposure to 0.2% and 0.5% ricinine for different durations. Data are presented as mean ± SEM from three independent experiments. Asterisks (*p* < 0.05) denote significant differences compared to the control group, **p < 0.01.

To investigate the effects of ricinine on larval development, 2nd- and 3rd-instar larvae of three insect species were fed an artificial diet containing varying concentrations of the toxin. As shown in **Figure 1** and **Table 1**, *B. mori* larvae exhibited significant growth inhibition from day 1 to day 4 after feeding on diets containing 0.2% (w/w) and 0.5% (w/w) ricinine. Larvae in these treatment groups displayed reduced mobility and significantly increased mortality during the first four days (**Table 2**). Moreover, surviving larvae showed negligible weight gain after the initial day of exposure. In contrast, no mortality occurred in the ricinine-treated groups of either *S. cynthia ricini* or *S. litura*. However, ricinine exerted potent growth-inhibitory effects on *S. litura*: compared to the control group, the 0.5% treatment group exhibited a weight reduction exceeding 50% on days 3-4. A transient growth suppression was also observed in *S. cynthia ricini* between days 2 and 3, followed by rapid recovery by day 4. Thus, after normalization to the species-specific baseline and time-matched control, ricinine produced distinct species-dependent responses under the experimental conditions, including acute lethality in *B. mori*, sustained growth inhibition in *S. litura*, and transient growth suppression in *S. cynthia ricini*. Representative growth detail data of the three insect species are presented in **Supplementary Figure 1**.

**Table 1:** Changes in the body weights ofof *B. mori, S. litura* and *S. cynthia ricini* larvae after different times of 0.2% and 0.5% ricinine exposure.

Species	Day	Repeat 1			Repeat 2		
		Control (mg)	0.2% ricinine (mg)	0.5% ricinine (mg)	control (mg)	0.2% ricinine (mg)	0.5% ricinine (mg)
*B. mori*	0	11.3 ± 1.1	11.3 ± 2.1	11.0 ± 2.2	11.2 ± 1.5	11.4 ± 1.8	11.0 ± 1.8
	1	17.4 ± 2.1	10.4 ± 1.9^**^	9.5 ± 1.9^**^	15.6 ± 1.7	11.1 ± 1.9^**^	10.1 ± 1.8^**^
	2	17.6 ± 2.1	9.8 ± 1.8^**^	9.8 ± 2.2^**^	17.8 ± 1.8	11.2 ± 1.6^**^	10.3 ± 0.7^**^
	3	23.6 ± 3.6	11.6^**^	--	20.3 ± 3.0	11.6 ± 2.1^**^	10.5^**^
	4	35.6 ± 6.2	--	--	29.1 ± 5.0	12.4^**^	--
S. *litura*	0	22.7 ± 5.2	21.6 ± 4.6	23.8 ± 4.2	22.8 ± 5.2	20.5 ± 4.3	21.7 ± 4.0
	1	58.1 ± 10.7	49.7 ± 10.8	39.8 ± 6.9^**^	58.6 ± 11.1	40.9 ± 8.3^**^	41.0 ± 8.9^**^
	2	99.0 ± 21.1	77.7 ± 20.5^*^	61.7 ± 15.7^**^	99.7 ± 26.0	74.8 ± 20.7^*^	71.8 ± 13.0^**^
	3	194.9 ± 52.7	146.9 ± 43.7^*^	92.0 ± 23.7^**^	218.2 ± 34.3	129.5 ± 52.6^**^	93.8 ± 15.0^**^
	4	295.0 ± 81.3	252.0 ± 66.9	144.7 ± 43.7^**^	331.1 ± 57.0	225.2 ± 73.7^**^	153.2 ± 33.1^**^
*S. cynthia ricini*	0	7.7 ± 0.3	8.0 ± 0.6	8.1 ± 0.7	7.9 ± 0.5	8.0 ± 0.8	8.0 ± 0.7
	1	8.2 ± 0.6	8.6 ± 0.5	8.7 ± 0.6	7.9 ± 0.7	8.3 ± 1.1	8.7 ± 0.6
	2	18.3 ± 5.2	15.5 ± 4.0	15.0 ± 3.0	16.1 ± 2.7	10.4 ± 2.2^**^	13.2 ± 1.7^**^
	3	31.5 ± 4.0	26.0 ± 3.2^**^	25.6 ± 3.2^**^	35.7 ± 3.3	20.4 ± 3.8^**^	24.3 ± 3.2^**^
	4	40.9 ± 9.5	35.5 ± 8.9	36.6 ± 4.3	43.5 ± 13.0	33.5 ± 5.6^*^	34.0 ± 4.1

*p < 0.05 and **p<0.01 indicate significant differences and extremely significant differences, respectively, compared to the control group.

**Table 2:** Changes in mortality and growth-inhibition rate of *B. mori, S. litura* and *S. cynthia ricini* larvae after different times of 0.2% and 0.5% ricinine exposure.

Species	Day	Repeat 1 mortality/inhibiting rate (%)			Repeat 2 mortality/inhibiting rate (%)		
		Control	0.2% ricinine	0.5% ricinine	control	0.2% ricinine	0.5% ricinine
*B. mori*	1	0/-	30/40.2	0/45.4	0/-	0/28.8	10/35.3
	2	0/-	40/44.3	50/44.3	0/-	60/37.1	80/42.1
	3	0/-	90/50.8	100/-	0/-	70/42.9	90/48.3
	4	0/-	100/-	100/-	0/-	90/57.4	100/-
*S. litura*	1	0/-	0/14.5	0/18.3	0/-	0/30.2	0/30.0
	2	0/-	0/21.5	0/37.7	0/-	0/25.0	0/28.0
	3	0/-	0/24.6	0/52.8	0/-	0/40.7	0/57.0
	4	0/-	0/14.6	0/50.9	0/-	0/32.0	0/53.7
*S. cynthia ricini*	1	0/-	0/-0.5	0/-0.6	0/-	0/-0.5	0/-10.0
	2	0/-	0/15.3	0/18.0	0/-	0/35.4	0/18.0
	3	0/-	0/17.5	0/18.7	0/-	0/42.9	0/31.9
	4	0/-	0/13.2	0/10.5	0/-	0/23.0	0/21.8

### 3.2 Metabolomic analysis of hemolymph from *B. mori*, *S. cynthia ricini*, and *S. litura* following ricinine exposure

**Figure 2:**
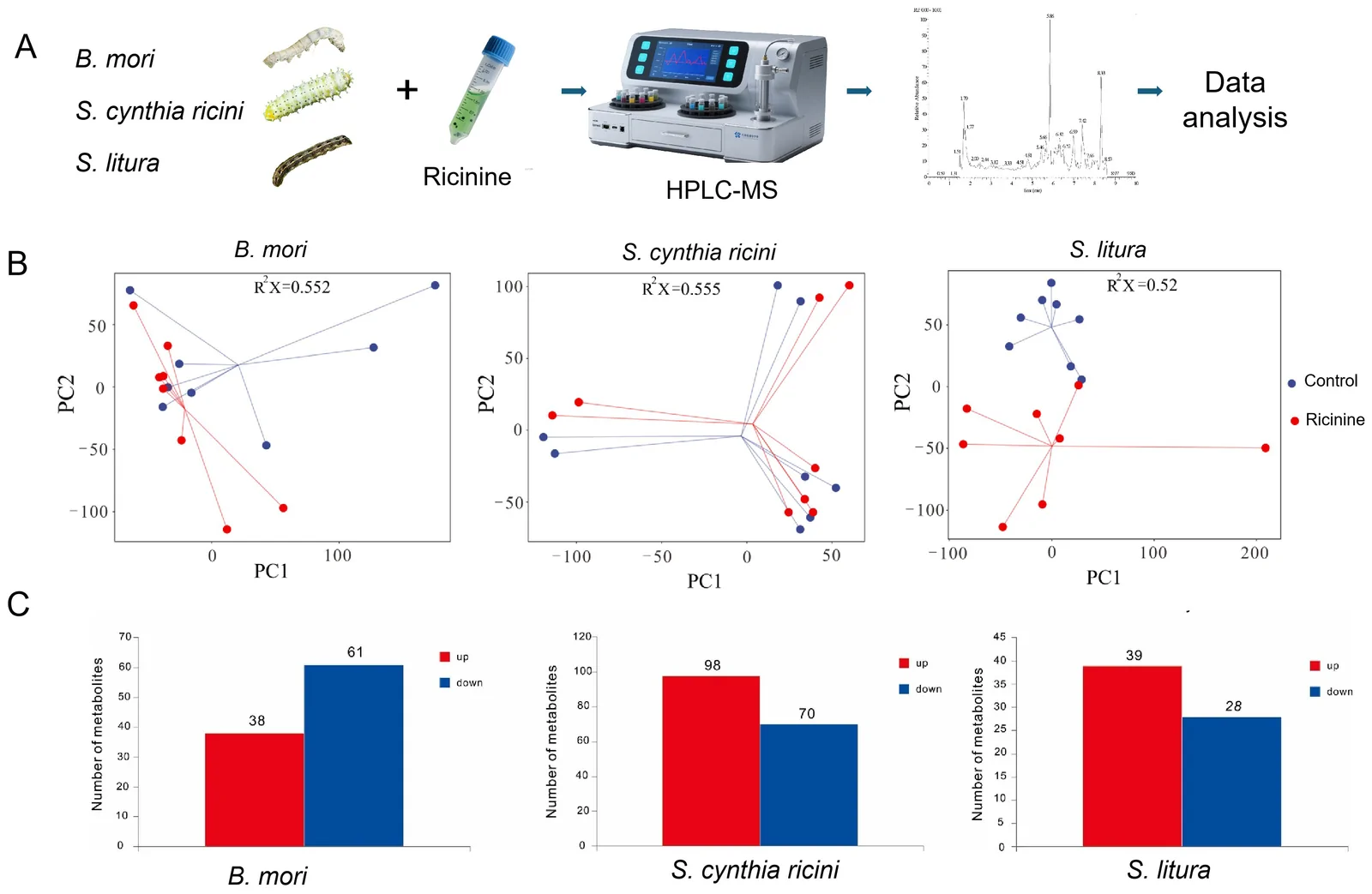
Analysis of alterations in the hemolymph metabolome of *B. mori*, *S. cynthia ricini*, and *S. litura* following 48 h of 0.5% ricinine exposure using HPLC-MS. **(A)** Schematic diagram of the metabolomics analysis workflow. **(B)** PCA plots of *B. mori*, *S. cynthia ricini*, and *S. litura* samples following ricinine exposure. Each point represents one biological sample (n = 8 larvae per treatment and species); blue and red points indicate control and ricinine-treated samples, respectively. PC1 and PC2 represent the first and second principal components, and the corresponding R^2^X value indicates the cumulative proportion of metabolomic variation explained by the displayed components. PCA was performed using metabolite peak areas normalized to a total signal of 10,000 for each sample. **(C)** Numbers of significantly increased (red) and decreased (blue) metabolites in each species following ricinine exposure. Bar height and the value above each bar indicate the number of differential metabolites.

To investigate ricinine-induced metabolic alterations, hemolymph from *B. mori*, *S. cynthia ricini*, and *S. litura*, was analyzed using HPLC-MS. In metabolomics analysis, the ricin-treated group of each species was compared with its respective control group at the same time point. Within each species, metabolomic data from larvae exposed to 0.5% ricinine for 48 h were compared with data from the corresponding species-matched, time-matched, ricinine-free baseline-control group (**Figure 2A**). Subsequent PCA performed separately within each species revealed clear separation between the metabolic profiles of the treatment and control groups. Specifically, in *S. litura*, the metabolic profiles were completely separated, with no overlap among the eight sample points from each group. In *B. mori*, the centroid lines of the toxin-treated and control groups exhibited only minimal overlap. In contrast, the metabolic profile of *S. cynthia ricini* exposed to 0.5% ricinine closely aligned with that of its control group, as evidenced by the near-complete intersection of their centroid lines. These within-species comparisons indicate that ricinine induced detectable metabolic perturbations in *B. mori* and *S. litura*, whereas the response of *S. cynthia ricini* was comparatively limited under the tested conditions (**Figure 2B**).

Within-species differential analysis revealed distinct patterns across the three species: 38 upregulated and 61 downregulated metabolites in *B. mori*, 98 upregulated and 70 downregulated in *S. cynthia ricini*, and 39 upregulated and 28 downregulated in *S. litura* (**Figure 2C**). Notably, the profound metabolic difference observed in *B. mori* suggests its particular sensitivity to ricinine. KEGG pathway enrichment analysis identified the most significantly enriched pathways as “Taurine and hypotaurine metabolism” in *B. mori*, “ATP-binding cassette (ABC) transporters” in *S. litura*, and “D-Amino acid metabolism” in *S. cynthia ricini* (**Supplementary Figure 2**). These distinct enrichment results further underscore the differential responses of the three insect species to ricinine.

### 3.3 Differential metabolite identification of three insects following ricinine exposure

Following systematic identification, relative quantification, and statistical analysis, a series of differential metabolites were identified in the three insect species (**Table 3**). Notably, ricinine was detected in the hemolymph of all three insect species following exposure, indicating that the toxin successfully penetrated the peritrophic membrane. The highest normalized relative concentration of ricinine was found in the *S. cynthia ricini*, suggesting that this species might possess a greater capacity for ricinine uptake or a reduced metabolic clearance rate.

**Table 3:** Metabolic pathways closely associated with the response to ricinine exposure in *B. mori*, *S. cynthia ricini*, and *S. litura*.

Pathways	Metabolites	*B. mori*		*S. litura*		*S. cynthia ricini*	
		Control	0.5% ricinine	Control	0.5% ricinine	Control	0.5% ricinine
Oxidative phosphorylation	Ricinine	0.05 ± 0.02	1.87 ± 0.48^**^↑	0.07 ± 0.11	3.05 ± 1.5^**^↑	0.49 ± 0.21	155.50 ± 85.02^s**^↑
	FMN	0.19 ± 0.01	0.17 ± 0.01^**^↓	——	——	0.15 ± 0.07	0.14 ± 0.08
	Adenine	1.34 ± 0.44	0.59 ± 0.34^**^↓	1.11 ± 0.26	0.79 ± 0.19^*^↓	——	——
	Riboside	0.58 ± 0.38	0.58 ± 0.35	1.9 ± 0.3	1.56 ± 0.24^*^↓	5.78 ± 3.52	6.28 ± 3.78
	Nicotinic acid (NA)	0.4 ± 0.09	0.3 ± 0.07^*^↓	——	——	0.12 ± 0.05	0.14 ± 0.06
	Phosphoric acid (PA)	0.34 ± 0.21	0.14 ± 0.05^*^↓	3.26 ± 1.15	3.47 ± 0.97	5.61 ± 4.56	8.57 ± 7.67
TCA cycle	Citric acid	1.93 ± 0.42	1.97 ± 0.31	732.67 ± 84.67	728.57 ± 118.59	677.35 ± 52.64	613.91 ± 109.15
	Malic acid	169.28 ± 29.83	144.02 ± 36.49	6.05 ± 1.34	7.02 ± 2.81	585.52 ± 119.03	493.88 ± 164.56
	Succinic acid	2.1 ± 1.04	1.44 ± 0.38	276.81 ± 9.15	322.84 ± 87.7	226.39 ± 30.84	222.01 ± 47.59
	Fumaric acid	4.5 ± 0.61	4.36 ± 0.24	4.37 ± 0.84	4.85 ± 0.59	71.86 ± 13.76	68.02 ± 22.12
	Oxaloacetic acid	2.32 ± 0.55	2.19 ± 0.6	0.53 ± 0.17	0.50 ± 0.15	0.23 ± 0.12	0.14 ± 0.04
Detoxification	Carboxylate	1.74 ± 0.3	2.29 ± 0.54^*^↑	4.2 ± 0.31	4.6 ± 0.4^*^↑	1.28 ± 0.84	0.92 ± 0.85
	Glutathione	——	——	0.1 ± 0.11	0.71 ± 0.47^**^↑	0.26 ± 0.16	0.2 ± 0.14
	Sulfate	8.25 ± 4.93	16.63 ± 7.76^**^↑	2.59 ± 1.16	3.06 ± 2.8	1.8 ± 0.75	1.65 ± 0.44
Amino acid metabolism	Threonine	16.46 ± 3.36	11.91 ± 2.56^**^↓	7.44 ± 0.93	5.96 ± 0.61^**^↓	15.35 ± 2.68	13.91 ± 3.29
	Histidine	146.49 ± 16.93	152.64 ± 21.58	194.8 ± 8.82	148.16 ± 29.97^**^↓	147.79 ± 51.1	166.22 ± 74.48
	Targinine	——	——	6.98 ± 1.02	5.07 ± 0.71^**^↓	13.03 ± 2.54	14.55 ± 4.5
	Serine	3.2 ± 0.99	5.43 ± 2.2^*^↑	25.39 ± 5.67	35.57 ± 6.86^**^↑	39.96 ± 14.54	48.66 ± 36.34
	Lysine	48.17 ± 10.09	44.78 ± 5.4	187.29 ± 23.31	150.77 ± 20.08^**^↓	101.2 ± 41.53	74.62 ± 39.37
	Ornithine	0.8 ± 0.69	0.43 ± 0.16	34.69 ± 14.85	19.48 ± 7.38^*^↓	86.4 ± 33.46	59.73 ± 19.63
	Valine	17.75 ± 8.5	27.19 ± 8.53^*^↑	1.03 ± 0.05	0.97 ± 0.06	1.01 ± 0.22	0.86 ± 0.22
	Glutamine	14.42 ± 2.67	14.9 ± 1.42	60.26 ± 7.82	72.36 ± 11.95^*^↑	181.88 ± 64.88	167.07 ± 86.64
Carbohydrates and others	Glucopyranoside	0.44 ± 0.36	0.057 ± 0.035^*^↓	7.13 ± 1.67	4.02 ± 0.87^**^↓	0.12 ± 0.1	0.47 ± 0.39^*^↑
	Sucrose	2.55 ± 0.98	4.20 ± 1.42^*^↑	0.95 ± 0.26	1.52 ± 0.45^**^↑		
	Ethyl phosphate	0.47 ± 0.22	0.13 ± 0.15^**^↓	0.56 ± 0.57	0.02 ± 0.02^*^↓	0.05 ± 0.01	0.05 ± 0.04
	Sn-glycero-3-phosphocholine	2.31 ± 0.66	0.45 ± 0.36^**^↓	6.21 ± 3.29	0.59 ± 0.38^**^↓	4.74 ± 1.28	3.89 ± 1.74
	Oxopentanoic acid	1.69 ± 1.24	421.10 ± 242.87^**^↑	5.14 ± 0.6	5 ± 1.09	26.05 ± 3.57	26.24 ± 4.21
	Creatine	0.33 ± 0.21	0.16 ± 0.05^*^↓	0.97 ± 0.41	1.09 ± 0.17	0.42 ± 0.11	0.48 ± 0.21
	Propanoic acid	0.14 ± 0.06	0.1 ± 0.03^*^↓	0.4 ± 0.08	0.26 ± 0.06^**^↓	0.37 ± 0.18	0.31 ± 0.21
	Prolinamide	1.62 ± 1.41	0.1 ± 0.06^**^↓	4 ± 1.76	2.2 ± 0.93^*^↓	9.68 ± 4.33	6.43 ± 2.49
	Fibrin	1.33 ± 0.36	0.92 ± 0.12^**^↓	——	——	——	——
	1-Deoxynojirimycin	107.57 ± 28.29	133.66 ± 13.07^*^↑	——	——	——	——
	Trigonelline	0.1 ± 0.04	4.08 ± 2.36^**^↑	——	——	——	——
	D-Glucose 6-phosphate	3.56 ± 1.52	1.32 ± 0.40^**^↓	——	——	——	——

Values are dimensionless normalized relative abundances calculated by setting the total LC-MS peak area of each sample to 10,000. All samples were prepared from an identical starting volume of hemolymph. ^*^p< 0.05 and ^**^ *p<*0.01 indicate significant differences and extremely significant differences, respectively, compared to the control group. The symbols ↑ and ↓ denote upward and downward trends, respectively, corresponding to the control group.

The normalized relative abundance of adenine levels were significantly decreased in both *B. mori* and *S. litura* compared to their respective controls. Adenine is related to purine and NAD biosynthesis, both of which are critical cofactors in metabolic processes. Furthermore, the normalized relative abundances of flavin mononucleotide (FMN) and phosphoric acid (PA) were significantly reduced in *B. mori* following ricinine treatment; both metabolites are essential components of the oxidative phosphorylation (OXPHOS) pathway. In contrast, carboxylate levels were significantly elevated in both *B. mori* and *S. litura*. Concurrently, glutathione levels were increased in *S. litura*, and sulfate levels were elevated in *B. mori*. Carboxylates, glutathione, and sulfate are key components of phase II detoxification systems in insects, and their accumulation suggests an activated physiological response to xenobiotic stress.

Ricinine ingestion also disrupted amino acid metabolism in both *B. mori* and *S. litura* larvae, as evidenced by decreased threonine levels, increased serine levels, and alterations in other amino acids. Additionally, dietary sucrose accumulated in both species, which may impede normal sucrose metabolism and consequently reduce the synthesis of carbohydrates such as glucopyranosides. Notably, ricinine ingestion induced significant changes in key metabolites, including ethyl phosphate (EP) and sn-glycero-3-phosphocholine (SNGPC), in both *B. mori* and *S. litura* larvae. Collectively, these metabolic alterations were associated with OXPHOS-related metabolism and may contribute to the impaired larval growth observed in *B. mori* and *S. litura*. This interpretation is consistent with the absence of significant changes in key TCA cycle metabolites—including citrate, malate, succinate, fumarate, and oxaloacetate—in all three insect species.

### 3.4 Transcriptome analysis of hemolymph from *B. mori*, *S. cynthia ricini*, and *S. litura* following ricinine exposure

**Figure 3:**
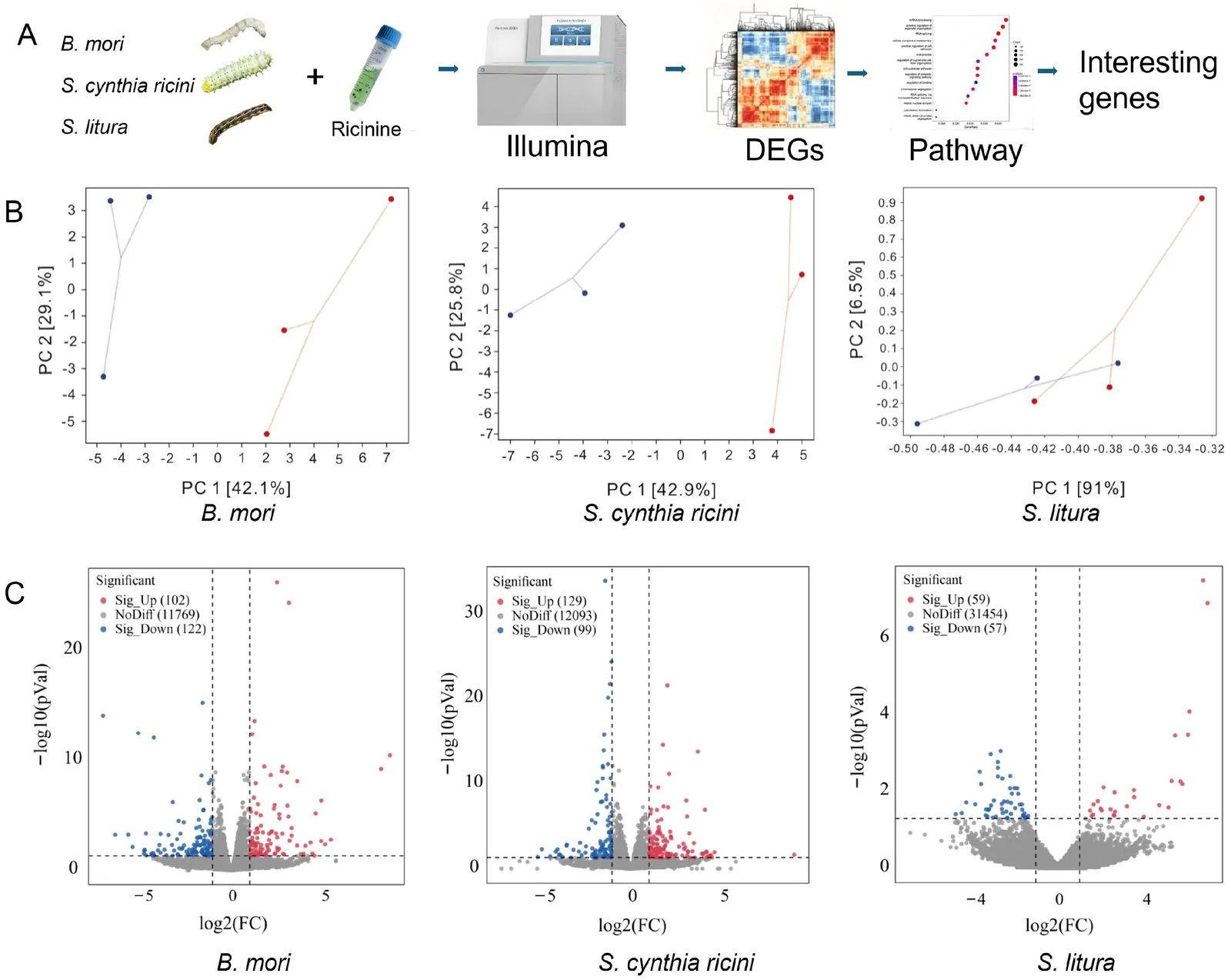
Transcriptome analysis of the hemolymph of *B. mori*, *S. cynthia ricini*, and *S. litura* following 48 h of 0.5% ricinine exposure using RNA-Seq. **(A)** Schematic workflow of the transcriptome analysis. **(B)** PCA plots of *B. mori*, *S. cynthia ricini*, and *S. litura* following ricinine exposure. Each point represents one biological replicate (n = 3 per treatment and species); blue and red points indicate control and ricinine-treated samples, respectively. PC1 and PC2 indicate the first and second principal components, with the percentages in parentheses showing the proportion of total transcriptomic variance explained by each component. **(C)** Volcano plots illustrating DEGs in *B. mori*, *S. cynthia ricini*, and *S. litura* following ricinine exposure. The x-axis represents log_2_FoldChange, and the y-axis represents -log_10_(*p*-value). Red, blue, and gray points indicate significantly upregulated, significantly downregulated, and non-significant genes, respectively. DEGs were defined as |log_2_FoldChange| >1 and adjusted *p* < 0.05; dashed lines indicate these thresholds. Numbers in parentheses indicate the number of genes in each category.

The RNA-Seq analysis of *B. mori*, *S. cynthia ricini*, and *S. litura* was performed following ricinine exposure, according to the schematic workflow shown in **Figure 3A**. After removing redundant reads, sufficient sequencing data were obtained for each group of all species (**Supplementary Table 2**). The base calling accuracy was high, with approximately 99% of bases achieving the Phred quality score of 20 (Q20; base-call accuracy ≥99%) and approximately 97% of bases achieving the Q30 standard (accuracy ≥ 99.9%). The guanine-cytosine (GC) content was consistent across all samples, ranging between 41% and 45%, preliminarily indicating good data quality. Genome annotation results showed that at least 85.94% of reads from each *B. mori* sample were mapped to the silkworm reference genome, while the mapping rate for *S. litura* was 91.01%. Furthermore, a *de novo* assembly of the *S. cynthia ricini* transcriptome was performed to enhance the analytical depth.

PCA of the data from the three insect species after ricinine exposure revealed distinct separation patterns. The data for *B. mori* and *S. litura* showed clear separation, whereas the separation for *S. cynthia ricini* was less pronounced (**Figure 3B**). Through comparative analysis, differentially expressed genes (DEGs) were identified following ricinine exposure. In *B. mori*, 224 DEGs were identified, comprising 102 up-regulated and 122 down-regulated genes. In *S. litura*, 228 DEGs were identified, with 129 up-regulated and 99 down-regulated genes. In *S. cynthia ricini*, 116 DEGs were identified, consisting of 59 up-regulated and 57 down-regulated genes (**Figure 3C**). Further KEGG pathway enrichment analysis revealed that the DEGs in *B. mori* were primarily enriched in the “Longevity regulating pathway - multiple species”. In *S. cynthia ricini*, DEGs were mainly enriched in the “Toll and Imd signaling pathway”, while in *S. litura*, they were predominantly enriched in the “Pentose and glucuronate interconversions” pathway (**Supplementary Figure 3**). These data indicate that, compared to *B. mori* and *S. litura*, ricinine treatment had a lesser impact on mRNA transcription in *S. cynthia ricini*.

### 3.5 DEGs identification of three insects following ricinine exposure

Based on transcriptome analysis, DEGs associated with detoxification, immune response, energy metabolism, digestion, and other processes were identified in the larvae of three insects following ricinine expose (**Table 4**). Among the detoxification enzymes, five major categories were identified, including cytochrome P450 monooxygenases (P450s), glutathione enzymes, glycosyltransferase, carboxylesterases, and ABC transporters. *In B. mori*, four genes were upregulated and four were downregulated. In *S. litura*, ten genes were upregulated and six were downregulated. With respect to immune responses, DEGs encompassed seven categories, including peptidoglycan recognition protein, glucan-binding protein, tetraspanin, cuticle protein, stromelysin, and serine protease inhibitors. In *B. mori*, five genes were upregulated and one was downregulated, whereas eight genes were upregulated in *S. litura*. In the context of energy metabolism, DEGs were classified into ten categories. In *B. mori*, four genes were downregulated. Conversely, in *S. litura*, two genes were upregulated and five were downregulated. Additionally, DEGs encoding digestive enzymes for carbohydrates, lipids, and protein digestion were represented by eleven categories. In *B. mori*, three genes were upregulated and ten were downregulated. In *S. litura*, nine genes were upregulated and six were downregulated.

**Table 4:** Enrichment pathways of DEGs closely associated with the response to ricinine exposure in *B. mori*, *S. cynthia ricini*, and *S. litura*.

Gene group	Gene description	*B. mori*		*S. litura*	
		Up	Down	Up	Down
Detoxification enzymes	Cytochrome P450	1	1	5	2
	Glutathione enzymes	0	2	2	1
	Glycosyltransferase	1	0	0	1
	Carboxylesterase	1	0	0	1
	ABC transporters	1	1	3	1
Immune response	Lysozyme	0	0	3	0
	Peptidoglycan recognition protein	0	0	1	0
	Glucan-binding protein	0	0	1	0
	Tetraspanin	0	1	0	0
	Cuticle protein	4	0	1	0
	Stromelysin	0	0	1	0
	Serine protease inhibitors	1	0	1	0
Carbohydrate digestion	Maltase	0	1	0	0
	Trehalose transporter	2	3	1	1
Lipid digestion	Lipase	0	0	0	1
	Phospholipase	0	1	0	0
	Cholinesterase	0	0	0	2
	Phosphate acyltransferase	0	1	0	0
	Fatty acid synthase	0	0	1	0
Protein digestion	Trypsin	0	1	1	0
	Carboxypeptidase	1	0	2	1
	Aminopeptidase	0	2	2	0
	Serine Protease	0	1	2	1
Energy metabolism	Cytidylate kinase	0	1	0	0
	Phosphofructo kinase	0	0	0	1
	Lactate dehydrogenase	0	1	0	0
	Pyrophosphate synthase	0	0	1	0
	Purine/uridine nucleoside phosphorylase	0	0	1	1
	Malate dehydrogenase	0	1	0	0
	glyoxylate reductase	0	1	0	0
	acetyl-coenzyme A synthetase	0	0	0	1
	acetaldehyde oxidase	0	0	0	1
	carbonyl reductase [NADPH] 3	0	0	0	1

These transcriptional profiles were consistent with a stronger detoxification- and immune-associated response in *S. litura* than in *B. mori*. Furthermore, ricinine was associated altered expression of genes involved in NAD/NADH-linked energy metabolism, which may contribute to the significant growth retardation observed in both *B. mori* and *S. litura* larvae. It should be noted that a substantial number of DEGs from the *de novo* transcriptome assembly of *S. cynthia ricini* could not be functionally annotated; consequently, no genes related to the discussed metabolic or immune pathways were identified in this species. The transcriptome statistics of three species, corresponding National Center for Biotechnology Information (NCBI) accession numbers and log_2_FoldChange values for all DEGs are provided in **Supplementary Tables 2**, **S3**.

### 3.6 Analysis of the relationship between key candidate genes and ricinine exposure

**Figure 4:**
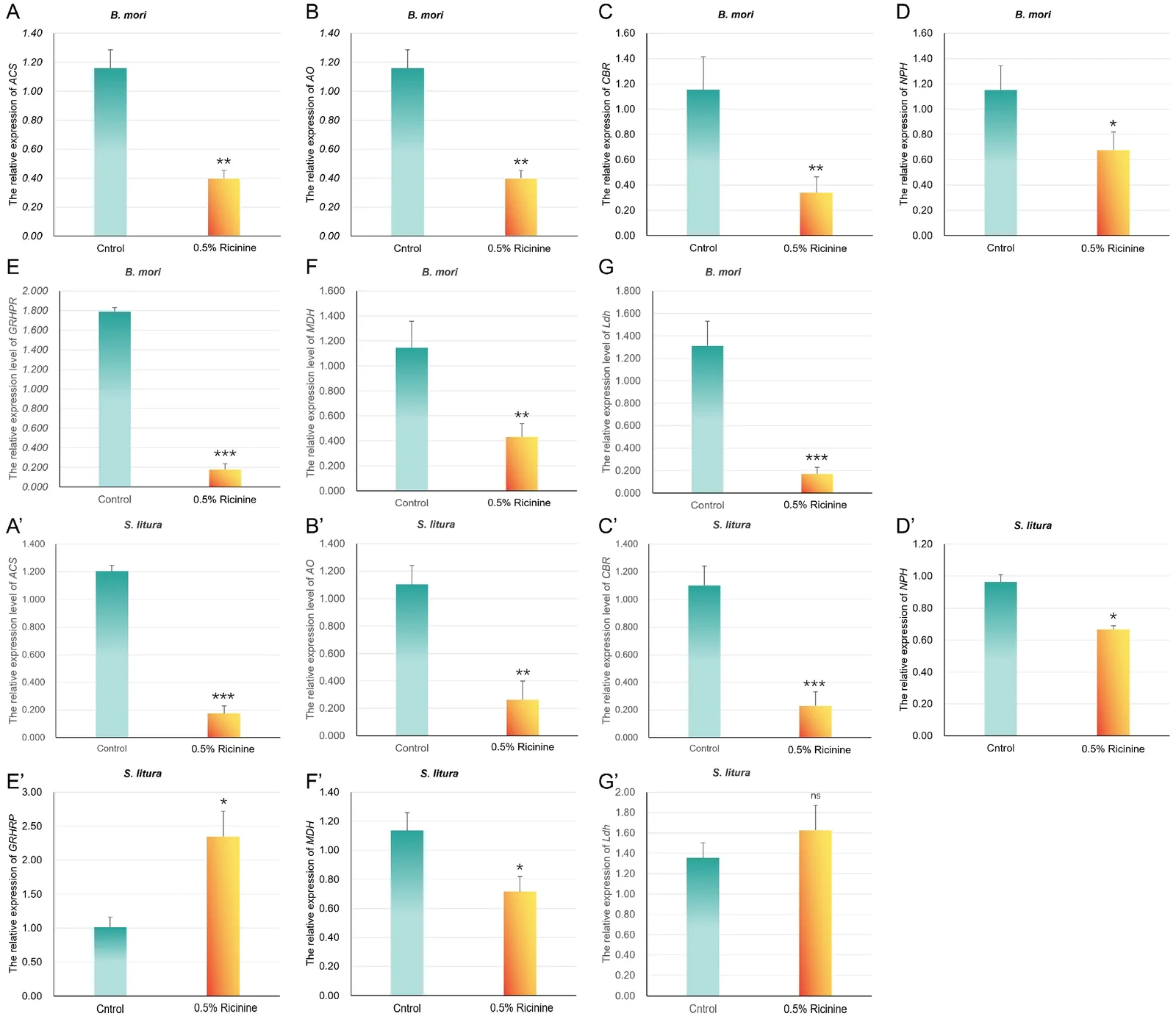
Analysis of the relative expression levels of seven key genes related to ricinine exposure in *B. mori* and *S. litura* using RT-qPCR. **(A–G)** *ACS*, *AO*, *CBR*, *NPH*, *GRHPR*, *MDH*, and *Ldh* expression in **(B)** mori following ricinine exposure. **(A’–G’)** *ACS*, *AO*, *CBR*, *NPH*, *GRHPR*, *MDH*, and *Ldh* expression in *S. litura* following ricinine exposure. The x-axis indicates treatment, and the y-axis indicates relative transcript abundance calculated using the 2^-ΔΔCt^ method. Expression was normalized to the species-specific *GAPDH* reference gene, and the corresponding control value was used as the calibrator. Data were normalized using the internal reference gene *BmGAPDH*, with a mean ± SD of three independent biological replicates. Significant differences are indicated by asterisks (*p* < 0.05). **p < 0.01, ***p < 0.001. ns, no significant difference.

Integrated metabolomic and transcriptomic analyses revealed that dietary supplementation with 0.5% ricinine induced substantial metabolic alterations in larvae of both *B. mori* and *S. litura*. Coordinated changes were observed in metabolites and transcripts associated with *β*-NAD-related metabolism and OXPHOS-associated processes. The expression levels of *NPH*, *lactate dehydrogenase* (*LDH*), *MDH*, *glyoxylate reductase/hydroxypyruvate reductase* (*GRHPR*), *carbonyl reductase* (*CBR*), *ACS*, and *aldehyde oxidase* (*AO*) were significantly perturbed (**Figure 4**). RT-qPCR validation showed consistent downregulation of *NPH*, *ACS*, *AO*, and *MDH* transcripts in ricinine-treated larvae of both species. Meanwhile, the transcript levels of *CBR*, *GRHPR*, and *LDH* were decreased in ricinine-treated *B. mori*. In contrast, *GRHPR* was significantly upregulated in ricinine-treated *S. litura*. The concordance between the sequencing and RT-qPCR data confirms the reliability of our omics findings. Furthermore, the coordinated changes in these genes, which participate in NAD/NADH-linked reactions and energy metabolism, support the involvement of *β*-NAD-related metabolic processes in the insect response to ricinine.

### 3.7 Analysis of β-NAD ‘s role in ricinine toxicity to the larvae of *B. mori* and *S. litura*

**Figure 5:**
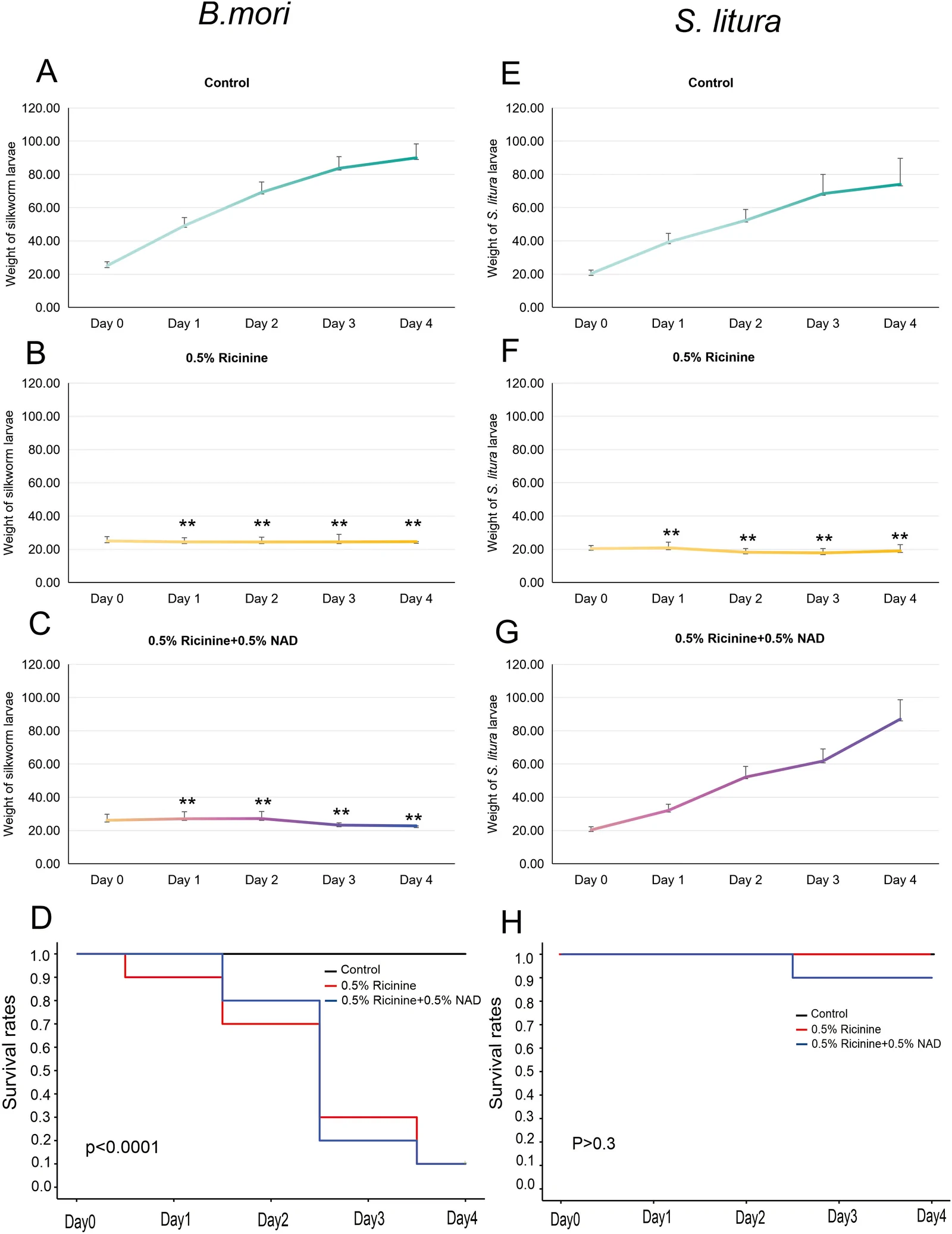
Analysis of the function of *β*-NAD in *B. mori* and *S. litura* in response to ricinine exposure. The weight of *B. mori* larvae without treatment **(A)**, after ricinine exposure **(B)**, and treatment with ricinine + *β*-NAD **(C)**. The weight and death of *S. litura* larvae without treatment **(E)**, after ricinine exposure **(F)**, and treatment with ricinine + *β*-NAD **(G)**. The x-axis indicates time after treatment, and the y-axis indicates larval body weight (mg). The Log-rank survival curves of *B. mori* and *S. litura* larvae after exposure to ricinine and ricinine+*β*-NAD for different durations **(D, H)**. Data are shown as mean ± SD of three independent biological replicates. Significant differences are indicated by asterisks (*p* < 0.05). **p < 0.01.

To elucidate the role of *β*-NAD in the insect response to ricinine, *B. mori* and *S. litura* were selected as model organisms for bioassays. Larvae were fed diets supplemented with either 0.5% ricinine alone or 0.5% ricinine combined with 0.5% *β*-NAD. Body weight and mortality were recorded at various time points post-administration. In *B. mori*, ricinine exposure significantly suppressed larval weight gain from day 1 to 4 and induced high mortality, exceeding 90% by day 4 in both treatment groups. In contrast, dietary *β*-NAD supplementation alleviated the ricinine-induced growth and developmental inhibition in *S. litura* larvae. These results provide functional support for the involvement of *β*-NAD-related metabolic processes in the growth-inhibitory effect of ricinine on *S. litura*. Conversely, the ricinine-induced lethality in *B. mori* was not rescued by exogenous *β*-NAD (**Figure 5**). Representative larval growth phenotypes are documented in **Supplementary Figure 4**.

### 3.8 Effects of combined ricinine and AcMNPV treatment on *S. litura*

**Figure 6:**
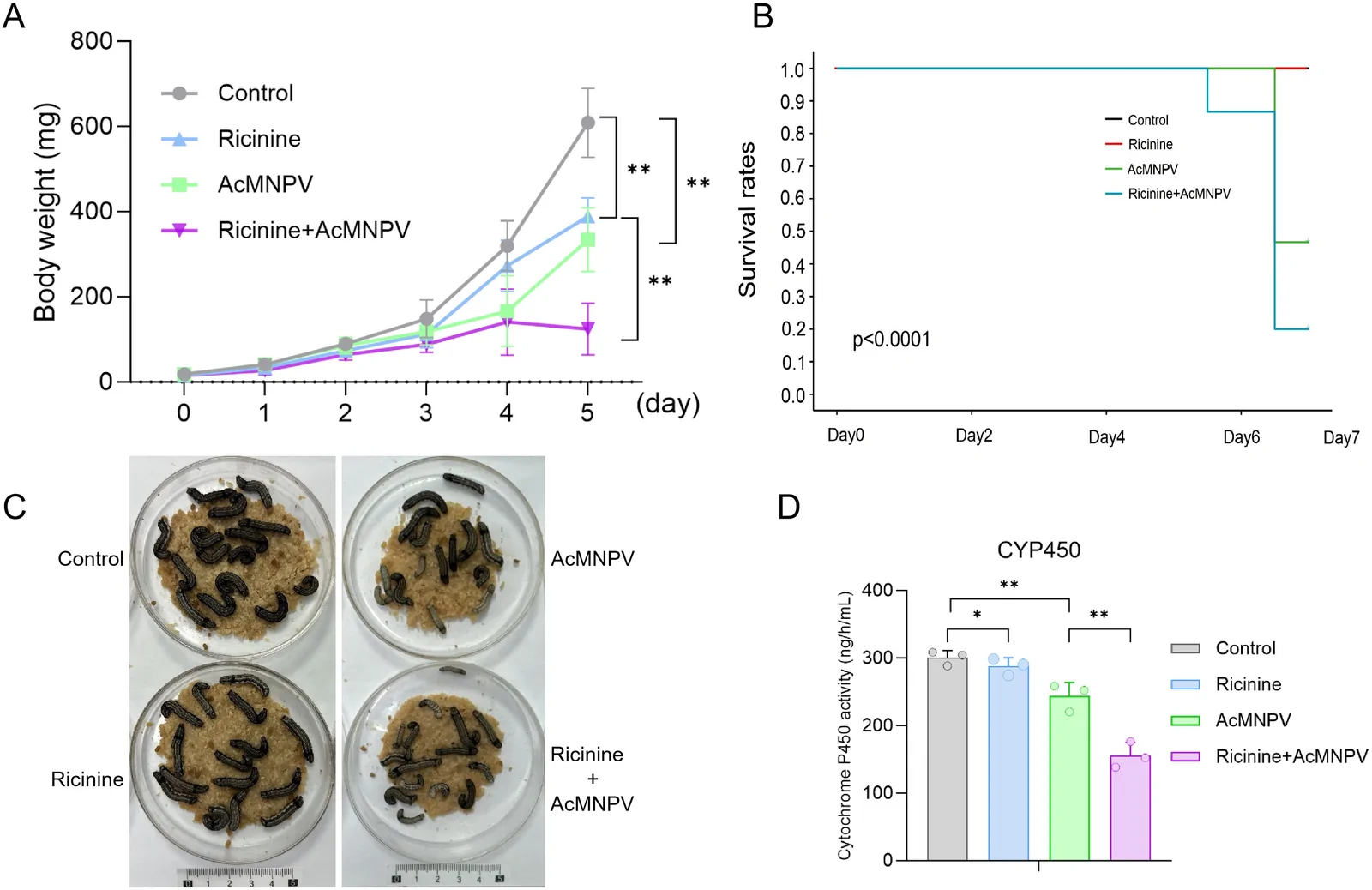
Effects of combined treatment with ricinine and AcMNPV on *S. litura*. **(A)** Changes in body weight of *S. litura* larvae from day 0 to day 5 following combined treatment with ricinine and AcMNPV. The x-axis indicates time after treatment (days), and the y-axis indicates body weight (mg). **(B)** Survival rates of *S. litura* after combined treatment with ricinine and AcMNPV. **(C)** Larval status on day 4 post-treatment with ricinine and AcMNPV. **(D)** Analysis of CYP450 activity following combined treatment with ricinine and AcMNPV. The y-axis indicates enzyme activity (ng/mL). Gray, blue, green, and purple symbols represent the control, ricinine, AcMNPV, and ricinine + AcMNPV groups, respectively. Data are presented as mean ± SEM from three independent experiments. Asterisks indicate significant differences compared to the control group (*p < 0.05, **p< 0.01).

To elucidate the role of ricinine in biological control, the combined effect of ricinine and AcMNPV on *S. litura* larvae was analyzed. The results showed that ricinine alone did not directly cause larval mortality but significantly inhibited their growth and development (**Figures 6A, C**). However, the ricinine + AcMNPV group exhibited greater growth inhibition and mortality than the groups receiving either ricinine or AcMNPV alone (**Figures 6A–C**), demonstrating an enhanced effect under the single-dose conditions tested. Furthermore, analysis of the marker protein CYP450 activity revealed that the combination of ricinine and AcMNPV significantly reduced CYP450 activity (**Figure 6D**), which was associated with a marked increase in larval mortality.

## 4 Discussion

Ricin, a highly toxic type II ribosome-inactivating protein produced by the seeds of the same plant, is classified as a security-sensitive biological toxin and a major biothreat agent (Pohanka, 2026; Moshiri et al., 2016); however, ricin and the small-molecule alkaloid ricinine are chemically and mechanistically distinct (Abomughaid et al., 2024). Therefore, evidence concerning ricin toxicity cannot directly explain the insecticidal activity of ricinine. Previous mechanistic studies of other insecticidal compounds identified inhibition of choline acetyltransferase by omeprazole analogs (Wang et al., 2024) and inhibition of vesicular acetylcholine transport by alkylsulfones (Goodchild et al., 2024). In contrast to these cholinergic mechanisms, our comparative multi-omics results associate the response to ricinine with perturbation of *β*-NAD-related metabolism and OXPHOS-associated processes. The findings revealed a spectrum of species-specific responses, ranging from severe toxicity and broad metabolic perturbation in *B. mori* to inducible detoxification-associated responses in *S. litura* and comparatively limited metabolic and transcriptional disruption in *S. cynthia ricini*.

Insects in natural environments are often exposed to various exogenous toxins, including plant secondary metabolites and synthetic chemicals such as pesticides and herbicides (Itoh et al., 2018; Kline and Joshi, 2024; Kshatriya and Gershenzon, 2024). Previous studies have distinguished symbiont-mediated degradation from host-encoded metabolic detoxification, particularly cytochrome P450-mediated responses (Nauen et al., 2022; Peterson, 2024). The transcriptional pattern observed in *S. litura* is more consistent with the latter: ricinine exposure increased the expression of multiple host genes associated with CYPs, glutathione metabolism, and ABC transport. This alignment extends the established association between inducible P450 expression and insect adaptation to xenobiotics to the response to ricinine (Lu et al., 2021). In *S. litura*, the upregulation of 18 genes related to detoxification and immune response, along with increased expression of *CYPs*, *glutathione enzymes* and *Lysozymes*, was consistent with the activation of a putative defense response to ricinine (**Table 4**). Notably, this response differs from the immunosuppression reported in imidacloprid-exposed *Drosophila*, in which the Duox and Imd pathways were impaired (Chmiel et al., 2019; Khan and Rolff, 2025). Ricinine instead induced multiple immune-related transcripts in *S. litura*, indicating that the direction of the immune response to xenobiotic exposure varies with both the compound and insect species. This activation response was significantly stronger than in *B. mori*, which upregulated only 9 related genes under the same conditions. Thus, the survival of *S. litura* despite marked growth inhibition was associated with a stronger inducible detoxification- and immune-related response, whereas the weaker response in *B. mori* coincided with substantially greater mortality. In lepidopteran insects, OXPHOS is closely linked to *β*-NAD generation to meet their high energy demands and efficient metabolic activities (Wilmsen and Dzialowski, 2023; Dong et al., 2025). A previous meta-analysis identified broad transcriptional changes associated with oxidative stress, primarily from publicly available *Drosophila* datasets (Bono, 2021). The present results align with this general association between oxidative stress and transcriptional perturbation but reveal a different level of specificity: coordinated changes in NAD-associated metabolites and energy-metabolism genes occurred in susceptible *B. mori* and *S. litura* but were comparatively limited in *S. cynthia ricini*. This interspecific contrast links the metabolic response more closely to ricinine susceptibility than would a uniform, nonspecific oxidative-stress response. *β*-NAD plays a central role in redox reactions and energy production, and its depletion can lead to cellular dysfunction and growth inhibition (Migaud et al., 2024). Luengo et al. showed that cellular demand for NAD^+^ relative to ATP can determine the direction of carbon metabolism (Luengo et al., 2021). Consistent with the central metabolic role of NAD^+^, ricinine-treated *B. mori* and *S. litura* showed decreased relative abundances of adenine, FMN, and pyruvate together with altered expression of NAD-associated genes. Unlike the glycolytic reprogramming described by Luengo et al., however, the present response was associated with OXPHOS-related metabolites and genes, whereas TCA-cycle metabolites remained largely unchanged. Collectively, the coordinated metabolite and transcript changes associate ricinine exposure with perturbation of *β*-NAD-related and OXPHOS-associated processes. The partial alleviation of growth inhibition by exogenous *β*-NAD in *S. litura* further supports the contribution of *β*-NAD-related metabolism to the observed response (**Figure 5**).

The most intriguing finding was the remarkable tolerance of *S. cynthia ricini*. Despite accumulating the highest relative abundance of ricinine in its hemolymph, this species exhibited minimal metabolic or transcriptional disruption. Previous work has identified sequestration and metabolic detoxification as major adaptations through which specialist insects exploit chemically defended host plants (Zhang et al., 2024; Peng et al., 2025). This pattern differed from tolerance based simply on reduced uptake or rapid clearance, as *S. cynthia ricini* retained a high relative abundance of ricinine while maintaining stable metabolic and transcriptional profiles. Consequently, the tolerance mechanism was hypothesized to involve specific metabolic enzymes or transporters, and functional validation of candidate genes via RNA interference or CRISPR knockout was deemed necessary. Its phenotype is therefore more consistent with intrinsic physiological tolerance to accumulated ricinine than with exclusion of the compound. This contrast with the pronounced responses of *B. mori* and *S. litura* connects host specialization with the ability to maintain metabolic stability during ricinine exposure.

**Figure 7:**
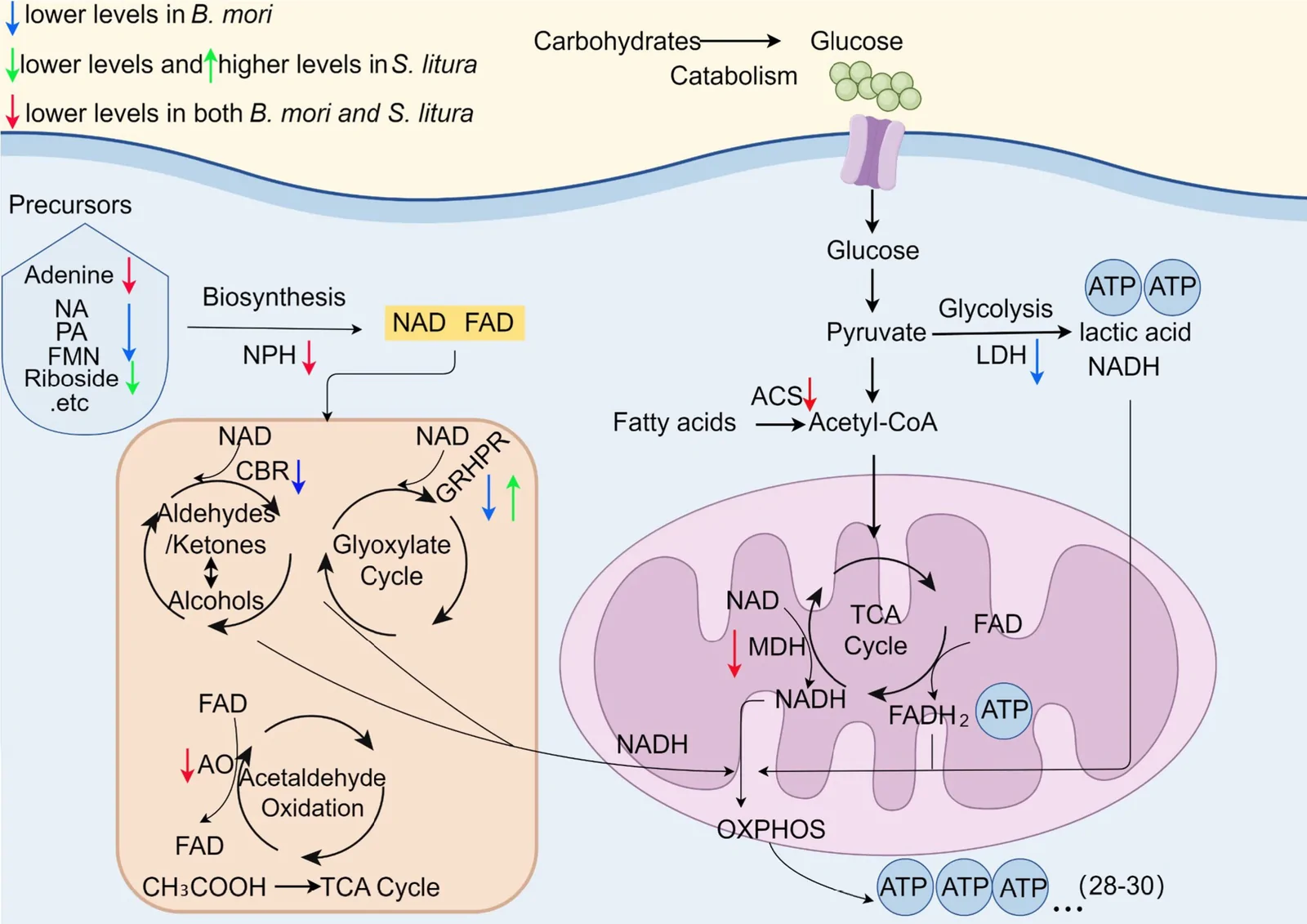
Proposed schematic model of the responses of *B. mori* and *S. litura* larvae to ricinine exposure. The observed responses suggest that ricinine may be associated with perturbations in NAD metabolism, while species-specific differences could potentially reflect variation in detoxification capacity and metabolic resilience. In *S. litura*, *β*-NAD supplementation partially alleviated ricinine-induced growth inhibition, suggesting that *β*-NAD-related metabolism contributes to the response to ricinine. Blue arrows indicate decreased metabolite or transcript levels in *B. mori*; green downward and upward arrows indicate decreased and increased levels, respectively, in *S. litura*; and red arrows indicate decreases shared by *B. mori* and *S. litura*. NAD, nicotinamide adenine dinucleotide; FAD, flavin adenine dinucleotide; FMN, flavin mononucleotide; ATP, adenosine triphosphate; TCA, tricarboxylic acid cycle; and OXPHOS, oxidative phosphorylation.

In summary, this study demonstrates that ricinine disrupts *β*-NAD metabolism and oxidative phosphorylation in lepidoptera, causing species-specific toxicity (**Figure 7**). From an applied perspective, the greater growth inhibition and mortality observed after combined ricinine and AcMNPV treatment suggest that this combination warrants further evaluation as a potential pest-control strategy. Future studies should therefore (i) directly quantify NAD^+^/NADH balance, ATP production, mitochondrial respiration, and related enzymatic activities; (ii) functionally validate candidate metabolic and detoxification genes using genetic or pharmacological approaches and clarify the tolerance mechanisms of S. cynthia ricini; and (iii) evaluate the efficacy, environmental persistence, resistance risk, and non-target safety of ricinine, alone or in combination with AcMNPV, before its potential application as a selective biopesticide.

## Data Availability

The original contributions presented in the study are publicly available. This data can be found here: https://www.ncbi.nlm.nih.gov/bioproject/PRJNA1516871/ and here: https://www.ncbi.nlm.nih.gov/bioproject/PRJNA1516910/.
