# Supplementary material for: Ricinine toxicity in susceptible lepidoptera involves perturbations in β-nicotinamide adenine dinucleotide metabolism
Source: Front Physiol. 2026 Sep 11;17:1947596. doi: 10.3389/fphys.2026.1947596 (PMC13612192; doi:10.3389/fphys.2026.1947596)
Supplement: Supplementary file 1 [file SupplementaryFile1.docx]

**Supplementary Figures**

**Figure S1.** Growth status of *B. mori*, *S. cynthia ricini*, and *S. litura* larvae on Day 2 (Corresponding to 3.1 in the manuscript).

**Figure S2.** KEGG enrichment analysis of differentially abundant metabolites (DAMs) in *B. mori*, *S. cynthia ricini*, and *S. litura* following ricin exposure. The bar chart displays enriched pathways grouped by functional categories across the three insect species. The y-axis represents pathway names, and the x-axis indicates the -log10(*p*-value) representing enrichment significance.

**Figure S3.** KEGG enrichment analysis of DEGs in *B. mori*, *S. cynthia ricini*, and *S. litura* following 48 h of ricin exposure. The bar chart displays enriched pathways grouped by functional categories across the three insect species. The y-axis represents pathway names, and the x-axis indicates the -log10(*p*-value) representing enrichment significance.

**Figure S4.** Typical growth status of *B mori* and *S. litura* larvae on Day 2 in the β-NAD supplementation experiment (Corresponding to Section 3.7 in the MS).
