# Supplementary material for: Ricinine toxicity in susceptible lepidoptera involves perturbations in β-nicotinamide adenine dinucleotide metabolism
Source: Front Physiol. 2026 Sep 11;17:1947596. doi: 10.3389/fphys.2026.1947596 (PMC13612192; doi:10.3389/fphys.2026.1947596)
Supplement: Supplementary file 2 [file SupplementaryFile2.docx]

**Supplementary Table S**

**Table S1**. The list of primer sequences used in RT-qPCR (Corresponding to Section 2.6 in the MS)

| Species | Gene names | Forward Primer (5′-3′) | Reverse Primer (5′-3′) |
| --- | --- | --- | --- |
| *B. mori* | *Lactate dehydrogenase* (*LDH*) | GGCCGCAGCTTTCTCTAT | CAGGTCCATCATCTCTCCTTTC |
|  | *Malate Dehydrogenase* (*MDH*) | CCGCCTCTGCACATGATAAA | ACGCCTTGCCTGTATTGTAG |
|  | *Glyoxylate Reductase* (*GRHPR*) | CGCTGTGGGTCTCATCATATC | ATCCATTGAACGCCGAACT |
|  | *Carbonyl Reductase* (*CBR*) | GCGAAATTGGTGACGGAAATAG | CCCTCCGCTCGCAATTAAA |
|  | *Acetyl-coenzyme A Synthetase* (*ACS*) | ATTTGTCGAGTCTCCGTGTG | CTCTTTCCCAATCCTGGTGTAG |
|  | *Acetaldehyde Oxidase* (*AO*) | GTCAACCTCCAAGCGTCTTAT | CGTCTAGTTCCACTTCAGCTATG |
|  | *nicotinate phosphoribosyltransferase (NPH)* | TAGAGTCGGCAATGGAATGG | GGAAAGGCTATGGCGTATGA |
|  | *β-Actin* (*Actb*) | GGCACCACACCTTCTACAAT | CATGATCTGGGTCATCTTCTCC |
| *S. Litura* | *Lactate dehydrogenase* (LDH) | GTTCCTACACACTACGCTGATG | CGCCAACTACAGTTACTCCTAAA |
|  | *Malate Dehydrogenase* (MDH) | GCTCCTTCCATTCCCAAAGA | TCTGTACTGGGACTCCAATCT |
|  | *Glyoxylate Reductase* (GRHPR) | CCTCAACTTTGGAGAGGAAGG | TTTGGAGATGGGATGGTGTG |
|  | *Carbonyl Reductase* (CBR) | ATAGCGTGGCCAAGTTTAGAG | GTAGGTACGGTCTTCGATGTTG |
|  | *Acetyl-coenzyme A Synthetase* (ACS) | CCTGACAATGACCGCTTCT | CACCATCTCGTCTCCGAATTTA |
|  | *Acetaldehyde Oxidase* (AO) | CTCTGCTCTCGTCTCCATCTA | GTGTTGATTCCTTGTCCCATTTC |
|  | *nicotinate phosphoribosyltransferase (NPH)* | GAGGAGTGGTCTGCTAAACTTC | CGCGTGCTAGTACAGACAAATA |
|  | *β-Actin* (Actb) | GAAGCCAGGTAAAGTGGTGCT | GTGTCCGTAGGGCTTGTCTG |

**Table S2**. Transcriptome statistics of *B. mori*, *S. cynthia ricini*, and *S. litura* following 48 h of ricin exposure. (Corresponding to Section 3.4 in the MS).

| Sample | Raw Reads No | Clean_Reads | GC(%) | Q20(%) | Q30(%) | Total_Mapped | Mapped_to_Gene | Mapped_to_InterGene | Mapped_to_Exon |
| --- | --- | --- | --- | --- | --- | --- | --- | --- | --- |
| *B. mori* Con1 | 48,263,140 | 47467682 | 42.53 | 98.71 | 96.59 | 41037276 (86.45%) | 36272445 (93.55%) | 2501794 (6.45%) | 34684531 (95.62%) |
| *B. mori* Con2 | 40,038,714 | 39420910 | 42.49 | 98.80 | 96.82 | 34137396 (86.60%) | 30031532 (93.54%) | 2072680 (6.46%) | 28787844 (95.86%) |
| *B. mori* Con3 | 39,897,052 | 39294132 | 42.72 | 98.80 | 96.80 | 33768258 (85.94%) | 29830086 (93.75%) | 1987838 (6.25%) | 28590652 (95.85%) |
| *B. mori* Ricin1 | 38,827,716 | 38208070 | 42.54 | 98.77 | 96.74 | 33116292 (86.67%) | 28865084 (93.57%) | 1982169 (6.43%) | 27698408 (95.96%) |
| *B. mori* Ricin2 | 44,106,188 | 43518686 | 42.37 | 98.88 | 97.00 | 37650055 (86.51%) | 33039240 (93.43%) | 2321987 (6.57%) | 31627888 (95.73%) |
| *B. mori* Ricin3 | 40,811,084 | 40251760 | 42.52 | 98.87 | 96.94 | 35143443 (87.31%) | 30566449 (94.42%) | 1806031 (5.58%) | 29611848 (96.88%) |
| *S. Litura* Con1 | 44,719,204 | 44099160 | 41.5 | 98.94 | 96.99 | 40314284 (91.42%) | 34441054 (89.49%) | 4043969 (10.51%) | 32813529 (95.27%) |
| *S. Litura* Con2 | 50,198,700 | 49681650 | 41.46 | 99.16 | 97.58 | 45623851 (91.83%) | 39160689 (89.91%) | 4393526 (10.09%) | 37479558 (95.71%) |
| *S. Litura* Con3 | 45,676,018 | 45189404 | 41.49 | 99.11 | 97.42 | 41478779 (91.79%) | 35301339 (89.57%) | 4108863 (10.43%) | 33773412 (95.67%) |
| *S. Litura* Ricin1 | 49,804,772 | 49185014 | 41.33 | 99.0 | 97.15 | 44932535 (91.35%) | 38109320 (89.19%) | 4620795 (10.81%) | 36337515 (95.35%) |
| *S. Litura* Ricin2 | 48,556,038 | 47979248 | 41.33 | 99.03 | 97.19 | 43668262 (91.01%) | 37079406 (89.12%) | 4525778 (10.88%) | 35152607 (94.80%) |
| *S. Litura* Ricin3 | 55,258,614 | 54618494 | 41.67 | 99.06 | 97.3 | 49947872 (91.45%) | 42781300 (89.85%) | 4831839 (10.15%) | 40912117 (95.63%) |
| *S. cynthia ricini* Con1 | 49769798 | 49145606 | 44.28 | 99.02 | 97.15 |  |  |  |  |
| *S. cynthia ricini* Con2 | 56860160 | 56231212 | 43.37 | 99.08 | 97.30 |  |  |  |  |
| *S. cynthia ricini* Con3 | 46956338 | 46327120 | 45.04 | 98.98 | 97.03 |  |  |  |  |
| *S. cynthia ricini* Ricin1 | 54086774 | 53430214 | 43.45 | 99.05 | 97.24 |  |  |  |  |
| *S. cynthia ricini* Ricin2 | 51679384 | 51023830 | 44.01 | 99.02 | 97.14 |  |  |  |  |
| *S. cynthia ricini* Ricin3 | 66073546 | 65275246 | 44.60 | 99.05 | 97.20 |  |  |  |  |

**Table S3**. The NCBI IDs and log2 (FC) values for the differentially expressed genes (Corresponding to Section 3.5 in the MS).

| Gene group | Gene description | *B. mori* | | *S. litura* | |
| --- | --- | --- | --- | --- | --- |
|  |  | NCBI ID | Log_2_ (FC) | NCBI ID | Log_2_ (FC) |
| Detoxification enzymes | Cytochrome P450 | 101742342  100036578 | 2.4615  -2.723 | 111354121  111358860  111355611  111359340  111356842  111353966  111356895 | 3.6528  -1.614  4.0396  1.4339  2.2096  2.9683  -3.881 |
|  | Glutathione enzymes | 101736877 | -1.684  -2.877 | 111353307  111360752  111360068 | -3.095  1.6943  3.1984 |
|  | Glycosyltransferase | 100500758 | 1.4315 | 111356736 | -1.297 |
|  | Carboxylesterase | 101746653 | 1.5036 | 111357585 | -1.317 |
|  | ABC transporters | 111357066  101746565 | 1.5036  1.0592 | 111351601  111360794  111361102  111357066 | -1.123  1.5956  1.0435  4.2884 |
| Immune response | Lysozyme |  |  | 111348452  111364585  111354109 | 1.5049  1.3214  2.0576 |
|  | Peptidoglycan recognition protein |  |  | 111355797 | 2.2072 |
|  | Glucan-binding protein |  |  | 111359124 | 2.0736 |
|  | Tetraspanin | 101739312 | -6.231 |  |  |
|  | Cuticle protein | 100379352  100379461  100379429  100379394 | 1.9361  3.9926  2.5835  1.6953 | 111353578 | 3.1875 |
|  | Stromelysin |  |  | 111353141 | 1.3762 |
|  | Serine protease inhibitors | 100190966 | 2.5031 | 111354037 | 1.5899 |
| Carbohydrate digestion | Maltase | 100500759 | -3.793 |  |  |
|  | Trehalose transporter | 111357328  101738007  101743817  101744355  101739796 | -4.146  -2.819  -1.487  1.1268  1.3233 | 111357328  111364461 | 1.4291  -1.571 |
| Lipid digestion | Lipase |  |  | 111350725 | -1.391 |
|  | Phospholipase | 101742098 | -3.137 |  |  |
|  | Cholinesterase |  |  | 111359444  111359657 | -1.502  -1.412 |
|  | Phosphate acyltransferase | 101741213 | -1.245 |  |  |
|  | Fatty acid synthase |  |  | 111359194 | 1.8403 |
| Protein digestion | Trypsin | 101735694 | -4.553 | 111355892 | 1.6614 |
|  | Carboxypeptidase | 101741626 | 2.6677 | 111352383  111352385  111353879 | -2.745  1.2427  1.6047 |
|  | Aminopeptidase | 101736877  100141459 | -1.684  -2.877 | 111360752  111360068 | 1.6943  3.1984 |
|  | Serine Protease | 101736544 | -2.029 | 111361084  111359956  111347872 | 1.3873  -1.196  1.7944 |
| Energy metabolism | Cytidylate kinase | 732890 | -1.005 |  |  |
|  | Phosphofructo kinase |  |  | 111364997 | -1.228 |
|  | Lactate dehydrogenase | 100124423 | -1.594 |  |  |
|  | Pyrophosphate synthase |  |  | 111348648 | 2.6198 |
|  | Purine/uridine nucleoside phosphorylase |  |  | 111364810  111358893 | -1.047  2.0894 |
|  | Malate dehydrogenase | 100101182 | -1.087 |  |  |
|  | Glyoxylate reductase | 101744454 | -1.143 |  |  |
|  | Acetyl-coenzyme A synthetase |  |  | 111360723 | -1.238 |
|  | Acetaldehyde oxidase |  |  | 111356424 | -1.461 |
|  | Carbonyl reductase [NADPH] 3 |  |  | 111352744 | -1.234 |
